# Association between serum lipoprotein(a) and mildly reduced eGFR: a cross-sectional study

**DOI:** 10.1186/s12882-023-03417-6

**Published:** 2023-12-08

**Authors:** Hong Zhang, Rui Chen, Shoukui Xiang, Pei Gao, Jing Zhu, Long Wang, Xiaohong Jiang, Fei Hua, Xiaolin Huang

**Affiliations:** 1 Department of Endocrine and Metabolic Diseases, The First People’s Hospital of Changzhou, Third Affiliated Hospital of Soochow University, 185 Juqianjie Road, Changzhou, Jiangsu 213000 China; 2 Department of Endocrine and Metabolic Diseases, The First People’s Hospital of Hefei, Third Affiliated Hospital of Anhui Medical University, Hefei, Anhui 230000 China

**Keywords:** Lipoprotein(a), Cardiovascular disease, Mildly reduced eGFR, Cross-sectional study

## Abstract

Lipoprotein(a) [Lp(a)] is a risk factor for cardiovascular disease (CVD) and aortic stenosis. However, the data on the relationship between Lp(a) and mildly reduced estimated glomerular filtration rate (eGFR) has been disputed. This study was conducted to assess the relationship between Lp(a) concentrations and mildly reduced eGFR in healthy subjects.This community-based, cross-sectional study enrolled 1,064 volunteers aged ≥ 40 years who lived in Yonghong Community, Zhonglou District, Changzhou, China, between December 2016 and December 2017. A mildly reduced eGFR was defined as eGFR between 60 and 90 mL/min/1.73m^2^. A standardized questionnaire and biochemical measurements were used to gather information about participants. The serum concentration of Lp(a) was determined using the latex-enhanced immunoturbidimetric test. Of the total study population, 34.8% (*n* = 370) were men, and the mean age was 66.8 ± 8.5 years. A significant association existed between Lp(a) levels and the risk of mildly reduced eGFR. Individuals with the highest tertile of Lp(a) had higher odds of mildly reduced eGFR after adjusting for various confounders (adjusted odds ratio [OR]: 1.80, 95% confidence interval [CI]: 1.24–2.60, *P* = 0.0025) compared to those with the lowest tertile of Lp(a). Multivariable logistic regression of studies in which Lp(a) was presented as continuous variables showed consistent results (adjusted OR: 1.23 for 1-SD increment of Ln-Lp(a), 95% CI: 1.05–1.43). Subgroup analyses showed that study characteristics such as age, sex, obesity, diabetes, and hypertension status did not significantly affect the association (*P* for all interactions > 0.05). These results suggest that higher serum Lp(a) level was an independent risk factor for mildly reduced eGFR.

**Supplementary Information:**

The online version contains supplementary material available at 10.1186/s12882-023-03417-6.

## Introduction

Chronic kidney disease (CKD) is a global public health problem, affecting 10% of the adult population worldwide [1]. The prevalence of CKD increases annually; according to the systematic analysis of the 2017 Global Burden of Disease Study, the prevalence of CKD increased by 29.3% from 1990 to 2017 [2]. The adverse effects of CKD include not only progression to end-stage renal disease but also an increased risk of all-cause mortality and cardiovascular disease (CVD) including congestive heart failure, stroke, myocardial infarction (MI), and peripheral artery disease [3–5]. Thus, early identification and prevention of CKD are crucial. In a national survey, a mildly reduced estimated glomerular filtration rate (eGFR), defined as 60–89 mL/min/1.73m^2^ according to the KDIGO 2012 Clinical Practice Guideline for the Evaluation and Management of Chronic Kidney Disease [3, 6], was regarded as the early stage of CKD [7, 8]. Previous studies have demonstrated that patients with mildly reduced eGFR were more likely to develop CKD compared to those with a normal eGFR [9, 10]. Furthermore, the Framingham Heart Study found that patients with a mildly reduced eGFR had higher incident risks of CVD and CKD than individuals with eGFR ≥ 90mL/min/1.73m^2^ [11]. Therefore, determining the possible risk factors for mildly reduced eGFR is important to better control its complications.

Lipoprotein(a) [Lp(a)] is a macromolecular complex in plasma. It is composed of one molecule of a low-density lipoprotein (LDL) particle containing apolipoprotein B-100 (apo B-100) and apolipoprotein(a) [apo(a)] [12]. Lp(a) is easily deposited on the vessel wall and affects the key characteristics of LDL moiety [13]. Due to its structural homology, Lp(a) can compete with plasminogen for fibrin binding sites and eventually inhibit the hydrolysis of fibrinogen [14]. Numerous previous studies have reported that Lp(a) is a risk factor for MI, ischemic stroke, coronary heart disease, and vascular and non-vascular mortality [15–18]. Lp(a) is also associated with an increased risk of reduced renal function and higher all-cause mortality in CKD patients [19, 20]. However, in the early stage of CKD, the association between higher Lp(a) and mildly reduced eGFR has not been detected in detail, and the conclusions are not consistent. The Penn Diabetes Heart Study (PDHS), a single-center observation cohort of type 2 diabetes patients, discovered that the probability of mildly reduced eGFR increased by 17% in the high Lp(a) (> 30 mg/dL) group [21]. The Third National Health and Nutrition Examination Survey (NHANES III) discovered no correlation between Lp(a) and mildly reduced eGFR [22]. Consequently, this cross-sectional study aimed to investigate the association between serum Lp(a) concentration and the early stage of CKD, described as mildly reduced eGFR in a well-defined community-based population.

## Materials and methods

### Study population

We conducted a cross-sectional study from December 2016 to December 2017. We recruited 1,328 permanent residents aged ≥ 40 years who had lived in the communities of Zhonglou District, Changzhou City, Jiangsu Province, China more than 6 months [23, 24]. We excluded those with missing information on Lp(a), creatinine, and serum cystatin C (Scys) and eGFR < 60 mL/min/1.73 m^2^ and deleted extreme values of Lp(a). Finally, 1,064 participants were included.

Each participant provided written informed consent. We collected information about the lifestyle and medical history of each participant via face-to-face interviews using a standard questionnaire. We collected a blood sample from each participant for biochemical measurements. The study protocol was approved by the Ethics Committee of the Third Affiliated Hospital of Soochow University.

### Data collection and biochemical measurements

Data on sociodemographic traits (such as age, sex, marital status, education, income level, and occupation), lifestyle factors (such as smoking, drinking, and daily physical activity), and personal medical histories (such as hypertension, diabetes, obesity, and other disorders, as well as the use of medications) were collected via face-to-face interviews. Current drinkers and smokers were defined as individuals who had consumed alcohol once every week, or at least one cigarette per day or seven cigarettes per week, for at least six months, respectively. We adopted the validated International Physical Activity Questionnaire (IPAQ) to collect detailed information about physical activity (intensity, frequency, and duration) and categorized participants as high physical activity or not.

Anthropometric measurements were performed by well-trained medical staff according to standardized protocols. Body weight and height were measured with the participants wearing light clothes without shoes to the nearest 0.1 kg and 0.1 cm, respectively. Body mass index (BMI) was calculated as weight divided by the square height (kg/m^2^). An automated electronic device (Omron Model HEM-752 FUZZY, Omron Company, Dalian, China) was used to automatically monitor blood pressure on the non-dominant arm while the participant was seated. This was performed three times in a row following at least a 5-min quiet rest, with a 1-min break between each measurement. Average systolic blood pressure (SBP) and diastolic blood pressure (DBP) were calculated using up to three readings for the analyses.

Venous blood samples were collected in the morning following a > 10-hour overnight fast. Levels of total cholesterol (TC), triglyceride (TG), high-density lipoprotein cholesterol (HDL-c), low-density lipoprotein cholesterol (LDL-c), aspartate aminotransferase (AST), alanine aminotransferase (ALT), serum cystatin C (Scys), serum creatinine (Scr), and uric acid (UA) were measured with an AU-5800 Chemistry System (Beckman, USA). Additionally, the Beckman Glucose Analyzer’s glucose oxidase method was used to measure fasting plasma glucose (FPG), and an automatic biochemical analyzer was used to perform a high-sensitivity latex-enhanced immunoturbidimetric test to detect the level of Lp(a). The eGFR was calculated according to the CKD-EPI formula for incorporating Scr and Scys: [25] eGFR = 135 × min(Scr/κ, 1)^α^ × max(Scr/κ, 1)^−0.601^ × min(Scys/0.8, 1)^−0.375^ × max (Scys/0.8, 1)^−0.711^ × 0.995^Age^ [×0.969 if female] [×1.08 if Black], where Scr is serum creatinine, Scys is serum cystatin C, κ is 0.7 for women and 0.9 for men, α is -0.248 for women and − 0.207 for men, min indicates the minimum of Scr/κ or 1, and max indicates the maximum of Scr/κ or 1.

### Definitions

#### Mildly reduced eGFR

Mildly reduced eGFR was defined as eGFR ≥ 60 mL/min/1.73 m^2^ and < 90 mL/min/1.73 m^2^.

#### Diabetes

Diabetes was defined as FPG ≥ 7.0 mmol/L (126 mg/dL) or diabetes previously diagnosed by a physician and treated with antidiabetic treatment.

#### Hypertension

Hypertension was defined as SBP ≥ 140 mmHg or DBP ≥ 90 mmHg or previously hypertension diagnosed by a physician and treated with anti-hypertensive drugs.

#### Obesity

Obesity was defined as BMI ≥ 28 kg/m^2^ according to the guidelines for the prevention and control of excess weight and obesity in Chinese adults [26].

### Statistical analysis

We divided the study participants into three groups according to the Lp(a) tertiles. Continuous variables in normal distributions were described as mean ± standard deviation (SD), and those in skewed distributions were described using median and interquartile range (IQR). Categorical variables were presented as numbers (proportions). TG, AST, ALT, and FPG were in skewed distributions and normalized using logarithmic transformation. We employed linear regression analyses for continuous variables and the Cochran–Mantel–Haenszel method for categorical variables to analyze the *P*-values for trends across the Lp(a) tertiles.

Multivariable-adjusted odds ratios (ORs) and their corresponding 95% confidence intervals (CIs) were calculated using multivariable logistic regression to evaluate the association between Lp(a) levels and the risk of mildly reduced eGFR with four models. All the confounders included in the multivariable logistic regression were selected based on the previous literatures. All the confounders were the risk factors of renal dysfunction and associated with Lp(a) levels, but not intermediate variables in the chain of Lp(a) and etiology of renal dysfunction. Confounders were classified into four groups: (1) Sociodemographic information; (2) lifestyle including smoking, drinking and physical activity; (3) metabolic-related factors including blood pressure, glucose, lipid profiles and liver enzymes; (4) serum uric acid which may be a critical risk factor of kidney dysfunction. Model 1 was adjusted for age, sex, and BMI. Model 2 was further adjusted for current smoking (yes/no), current drinking (yes/no), and physical activity based on Model 1. Model 3 was further adjusted for SBP, TG, TC, LDL-c, ALT, AST, and FPG based on Model 2. Model 4 was further adjusted for UA levels based on Model 3.

All analyses were performed using SAS version 9.4 (SAS Institute Inc, Cary, NC, USA), and bilateral *P*-values less than 0.05 were considered statistically significant.

## Results

### Characteristics of study population

The present study recruited 1,328 participants, We excluded those with missing information on Lp(a), creatinine, and serum cystatin C (Scys; *N* = 61) and eGFR < 60 mL/min/1.73 m^2^ (*N* = 181) and deleted extreme values of Lp(a) (*N* = 22). The total 1,064 individuals averagely aged 66.8 ± 8.5 years old, 34.8% of them were men. The median level of Lp(a) was 78 (35–134) mg/L, and the proportion of participants with mildly reduced eGFR was 52.44%. Table 1 shows the baseline characteristics of participants according to Lp(a) tertiles. BMI, ALT, AST, TG, and eGFR all tended to be lower in the higher compared with the lower Lp(a) tertile, but HDL-c and Scys both showed the opposite trend (*P* < 0.05). No statistically significant differences existed in age, DBP, SBP, Scr, FPG, UA, LDL-c, or TC (*P* > 0.05).

**Table 1:** Characteristics according to Lipoprotein(a) tertiles

Variables	Lipoprotein(a) tertiles			*P* for trend
	Tertile 1	Tertile 2	Tertile 3	
Numbers(n,%)	354 (32.27)	354 (33.27)	356 (33.46)	-
Lipoprotein(a)(mg/L)	26.0 (19.0–35.0)	77.5 (60.0–99.0)	186.0 (133.5-261.9)	< 0.0001
Age (years)	67.5 ± 8.1	65.9 ± 8.0	67.1 ± 9.2	0.54
Male (n,%)	120 (33.9)	130 (36.7)	120 (33.7)	0.96
BMI (kg/m^2^)	25.5 ± 3.6	25.9 ± 3.2	24.8 ± 3.5	0.009
Current smoker (n,%)	45 (12.82)	52 (14.73)	44 (12.39)	0.87
Current drinker (n,%)	13 (3.70)	29 (8.26)	17 (4.80)	0.53
High physical activity (n,%)	217 (61.3)	200 (56.5)	220 (61.8)	0.89
SBP(mmHg)	133.0 ± 13.9	134.0 ± 14.4	134.6 ± 15.3	0.15
DBP(mmHg)	82.3 ± 7.9	83.9 ± 9.0	82.6 ± 8.8	0.59
ALT (U/L)	20 (15–27)	19 (15–27)	18 (14–25)	0.02
AST (U/L)	23 (20–28)	22 (19–27)	22 (19–26)	0.04
TG (mmol/L)	1.79 (1.34–2.50)	1.61 (1.21–2.18)	1.42 (1.10–1.96)	< 0.0001
TC (mmol/L)	5.07 ± 0.98	5.09 ± 1.01	5.18 ± 0.96	0.14
HDL-c (mmol/L)	1.30 ± 0.29	1.33 ± 0.29	1.39 ± 0.31	< 0.0001
LDL-c (mmol/L)	2.69 ± 0.68	2.67 ± 0.69	2.76 ± 0.68	0.17
UA (µmol/L)	303.3 ± 69.6	301.8 ± 71.2	299.6 ± 72.0	0.49
FPG (mmol/L)	5.57 (4.69–6.73)	5.50 (4.86–6.87)	5.49 (4.79–6.72)	0.74
Scr (µmol/L)	73.21 ± 13.04	71.96 ± 12.08	73.22 ± 12.01	0.99
Scys (mg/L)	0.78 ± 0.14	0.83 ± 0.14	0.83 ± 0.15	< 0.0001
eGFR (mL/min/1.73m^2^)	90.14 ± 13.48	89.06 ± 13.51	87.30 ± 14.02	0.006
Hypertension (n, %)	242 (69.34)	222 (63.43)	241 (68.08)	0.73
Diabetes (n, %)	146 (41.95)	120 (34.19)	130 (37.14)	0.19

Data were means ± SD for normal variables, medians (interquartile ranges) for skewed variables, and numbers (percentages) for categorical variables

*P* values for trend were calculated by using linear regression analyses and Cochran-Armitage trend test for continuous and categorical variables across the three groups, respectively

*Abbreviations:* *BMI* Body mass index, *SBP* Systolic blood pressure, *DBP* Diastolic blood pressure, *ALT* Alanine aminotransferase, *AST* Aspartate aminotransferase, *TC* Total cholesterol, *TG* Triglycerides, *HDL-c* High-density lipoprotein cholesterol, *LDL-c* Low-density lipoprotein cholesterol, *UA* Uric acid, *FPG* Fasting plasma glucose, *Scr* Serum creatinine, *Scys* Serum cystatin C, *eGFR* Estimated glomerular filtration rate

### Association between Lp(a) and risk of mildly reduced eGFR

**Fig. 1:**
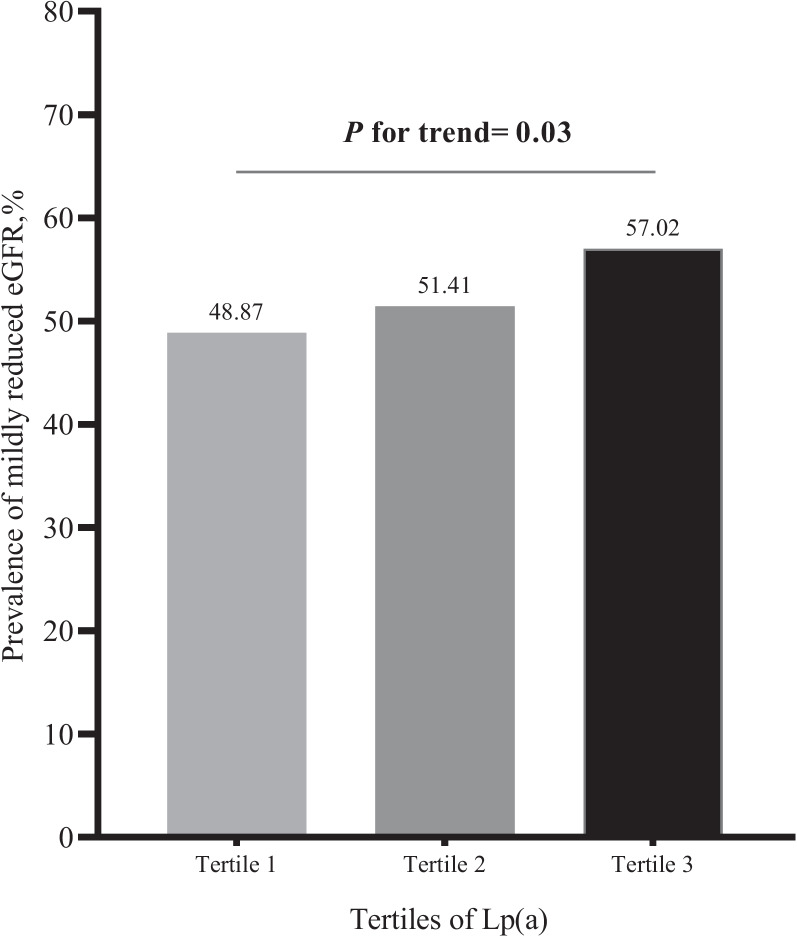
The prevalence of mildly reduced eGFR according to Lp(a) tertiles. *P* value for trend was calculated by using Cochran-Mantel-Haenszel method. Abbreviations: eGFR, estimated glomerular filtration rate; Lp(a), lipoprotein(a)

**Fig. 2:**
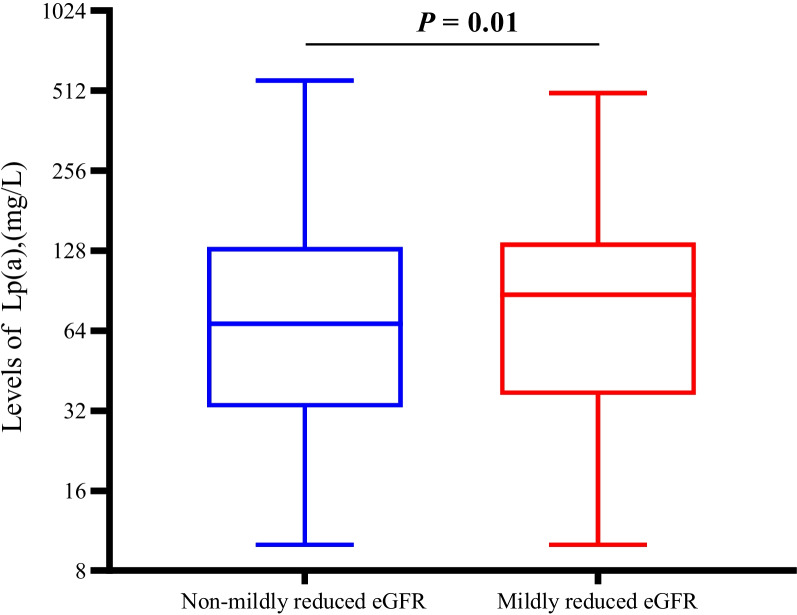
The level of Lp(a) in individuals with mildly reduced eGFR or not. *P* value was calculated by Generalized linear regression adjusting for age, sex, BMI, current smoker (yes / no), current drinker (yes / no), physical activity, SBP, ALT, AST, FPG, TC, TG, LDL-c and UA. Abbreviations: Lp(a), lipoprotein(a); eGFR, estimated glomerular filtration rate; BMI, body mass index; SBP, systolic blood pressure; ALT, alanine aminotransferase; AST, aspartate aminotransferase; FPG, fasting plasma glucose; TC, total cholesterol; TG, triglycerides; LDL-c, low-density lipoprotein cholesterol; UA, uric acid

Figure 1 shows the prevalence of mildly reduced eGFR according to Lp(a) tertiles. The prevalence of mildly reduced eGFR was 48.87%, 51.41%, and 57.02%, respectively (*P* for trend = 0.03), from the lowest to the highest tertiles of Lp(a). Figure 2 shows that those with mildly reduced eGFR had a higher Lp(a) compared to individuals without it (68 mg/L vs. 87.5 mg/L, *P* = 0.01).

We used multivariable logistic regression to evaluate the association between Lp(a) levels and the risk of mildly reduced eGFR. Table 2 shows that with the increment of Lp(a) tertiles, the risk of mildly reduced eGFR significantly increased (*P* for trend = 0.0025). In the age-, sex- and BMI-adjusted model, referenced to the lowest tertile, ORs in the second and highest Lp(a) tertiles were 1.41 (1.02–1.97) and 1.60 (1.14–2.23), respectively. Further adjusted for current smoking (yes/no), current drinking (yes/no), physical activity, SBP, TG, TC, LDL-c, ALT, AST, and FPG based on Model 1, 1.62- (OR = 1.62, 95% CI = 1.15–2.29) and 1.89-fold (OR = 1.89, 95% CI = 1.33–2.69) increases in the risk of mildly reduced eGFR were found in tertiles 2 and 3, respectively (*P* for trend = 0.0005). Adjusting for serum UA based on Model 3 did not significantly change the magnitude or direction of the association.The risk of mildly reduced eGFR, a continuous variable, increased by 27% (OR = 1.27, 95% CI = 1.10–1.47) with each 1-SD increment of ln-Lp(a) after adjusting for age, sex, BMI, current smoking (yes/no), current drinking (yes/no), physical activity, SBP, TG, TC, LDL-c, ALT, AST, and FPG. After further adjusting for serum UA, the OR was 1.23 (95% CI = 1.05–1.43).

**Table 2:** The association between the lipoprotein(a) and risk of mildly reduced eGFR

Lipoprotein(a) levels	OR (95%CI)			
	Model 1	Model 2	Model 3	Model 4
Tertile1	1.00(Ref)	1.00(Ref)	1.00(Ref)	1.00(Ref)
Tertile2	1.41(1.02–1.97)	1.42(1.02–1.99)	1.62(1.15–2.29)	1.68(1.17–2.41)
Tertile3	1.60(1.14–2.23)	1.62(1.16–2.26)	1.89(1.33–2.69)	1.80(1.24–2.60)
*P* for trend	0.0047	0.0038	0.0005	0.0025
Per SD increase in ln-Lp(a)	1.19(1.04–1.37)	1.20(1.05–1.37)	1.27(1.10–1.47)	1.23(1.05–1.43)

Model1: adjusted for age, sex, BMI

Model2: further adjusted for current smoker (yes/no), current drinker (yes/no) and physical activity based on Model1

Model3: further adjusted for SBP, TG, TC, LDL-c, ALT, AST, FPG based on Model2

Model4: further adjusted for UA based on Model3

*Abbreviations:* *BMI* Body mass index, *SBP* Systolic blood pressure, *ALT* Alanine aminotransferase, *AST* Aspartate aminotransferase, *TC* Total cholesterol, *TG* Triglycerides, *LDL-c* Low-density lipoprotein cholesterol, *UA* Uric acid, *FPG* Fasting plasma glucose

Additonally, we used the Youden index to calculate the cut-off point of Lp(a) for the diagnosis of mildly reduced eGFR (Supplemental Table 1). According to the Lp(a) cut-off point of 103 mg/L, participants were divided in two groups: elevated Lp(a) group (Lp(a) > 103 mg/L) and non-elevated Lp(a) group (Lp(a) ≤ 103 mg/L). Multivariable logistic regression was conducted to evaluate the association of Lp(a) with mildly reduced eGFR. After adjusting for age, sex, BMI, current smoking (yes/no), current drinking (yes/no), physical activity, SBP, TG, TC, LDL-c, ALT, AST, FPG and UA, elevated Lp(a) associated with 45% increased risk of mildly reduced eGFR (OR = 1.45, 95% CI = 1.07–1.97).

### Association of Lp(a) and mildly reduced eGFR stratified by age, sex, obesity, hypertension, and diabetes status

**Fig. 3:**
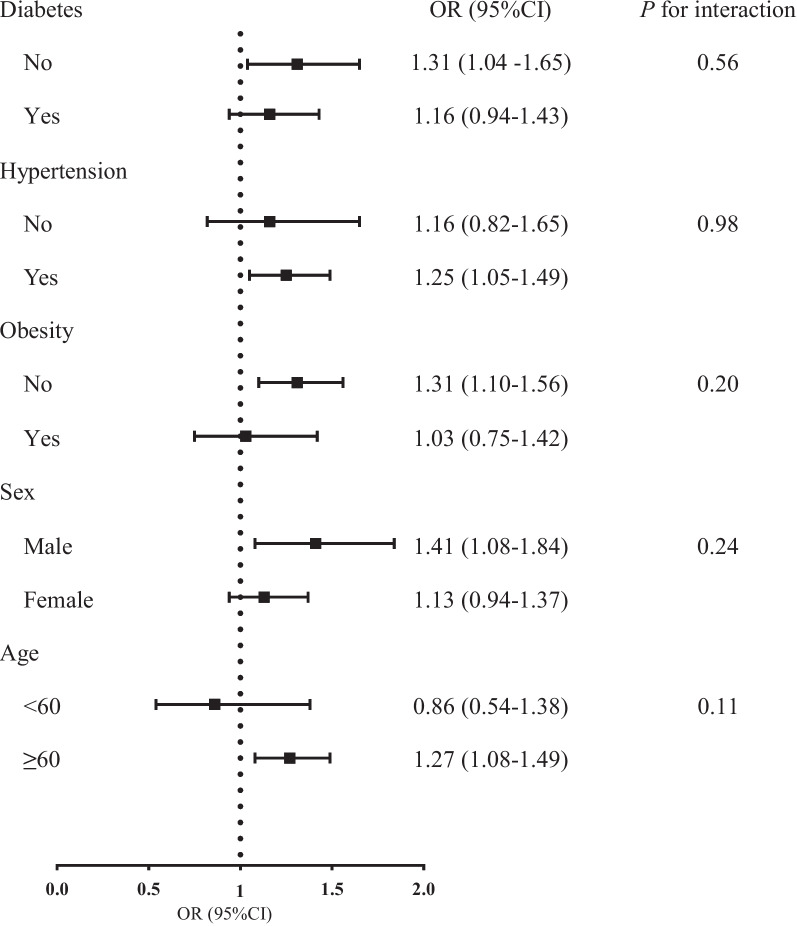
ORs of mildly reduced eGFR according to sex, age, obesity, hypertension and diabetes. ORs were calculated by multivariable logistic regression adjusting for age, sex, BMI, current smoker (yes / no), current drinker (yes / no), physical activity, SBP, ALT, AST, TC, TG, LDL-c, FPG and UA. Abbreviations: ORs, odds ratios; eGFR, estimated glomerular filtration rate; BMI, body mass index; SBP, systolic blood pressure; ALT, alanine aminotransferase; AST, aspartate aminotransferase; TC, total cholesterol; TG, triglycerides; LDL-c, low-density lipoprotein cholesterol; FPG, fasting plasma glucose; UA, uric acid

Figure 3 shows the associations between Lp(a) level and the risk of mildly reduced eGFR stratified by age (≥ 60/<60 years), gender (male/female), obesity (yes/no), hypertension (yes/no), and diabetes (yes/no). Age, sex, BMI, current smoking (yes/no), current drinking (yes/no), physical activity, SBP, TG, TC, LDL-c, ALT, AST, FPG, and UA were fully adjusted in the model. When we stratified the study participants by their major characteristics, we observed that the association of Ln-Lp(a) with mildly reduced eGFR risk was more evident among individuals who were male, older than 60 years, with hypertension, and without diabetes or obesity. The ORs when comparing those with mildly reduced eGFR to those with normal eGFR were 1.41, 1.27, 1.31, 1.25, and 1.31, respectively. However, the interaction effects of these factors with mildly reduced eGFR were not statistically significant (all *P* for interaction > 0.05).

## Discussion

In this community-based cross-sectional study enrolling 1,064 Chinese middle-aged and elderly participants, we found a significant association between Lp(a) levels and the risk of mildly reduced eGFR. With each increment in Lp(a) level, the risk of mildly reduced eGFR gradually increased, adjusting for various confounders. Furthermore, stratified analyses found that age, sex, obesity, diabetes, and hypertension status did not have a significant influence on the association between Lp(a) levels and the risk of mildly reduced eGFR.

At the end of the 20th century, Sechi et al. first found that the level of Lp(a) in patients with CKD was higher compared to that in participants without it and that the creatinine clearance rate gradually decreased as the Lp(a) decreased [27]. These results indicated that renal function is crucial in the catabolism of Lp(a) [28]. Recently, increasing evidence has indicated that an increase in Lp(a) plays an important role in the occurrence and development of renal function injury [19, 20]. A recent Mendelian randomized trial demonstrated an etiological association between serum Lp(a) levels and CKD; a 1-SD-reduced log10-Lp(a) level was associated with a 9% lower risk of CKD [29]. Another large prospective study with 10,375 middle-aged and elderly Chinese participants reported that after a mean follow-up of 4.4 years, each 1-unit increase in log10-Lp(a) was associated with a 1.99-fold (95% CI 1.15–3.43) increased risk of incident reduced renal function [19]. Additionally, a prospective cohort study conducted in South Korea enrolling 862 type 2 diabetic patients without CKD from Vincent’s Hospital found a significant association between the levels of Lp(a) and new CKD during 10.1 years of follow-up (HR = 2.12, 95% CI = 1.33–3.36) [30].

The majority of studies so far have focused on the relationship between Lp(a) and CKD, and no consensus exists about the association between Lp(a) and mildly reduced eGFR. The PDHS, a single-center observation cohort of type 2 diabetes patients, showed that the high-Lp(a) group had a 17% higher chance of having mildly reduced eGFR [21], whereas NHANES III found no association between Lp(a) and mildly reduced eGFR [22]. The discrepancy may be attributed to the sample size and study population. The present study found an increased risk of renal dysfunction in individuals with higher Lp(a) levels, consistent with the aforementioned cohort studies. The current study was the first to investigate the associations between Lp(a) and early renal dysfunction. It concluded that higher Lp(a) levels are closely related to an increased risk of mildly reduced eGFR in the middle-aged and elderly Chinese population. Our results also suggested that among individuals without CKD, Lp(a) might be a useful biomarker for the early detection of renal dysfunction. More novel biomarkers are needed to better assess risk stratification in CKD patients in the longitudual study [31].

The unique structure of Lp(a) may account for its ability to increase renal failure. Lp(a) includes apo B-100, a low-density lipoprotein-like fraction believed to promote atherosclerosis, and apolipoprotein A fraction, which is similar to plasminogen and may mediate an increased risk of thrombosis [32, 33]. Many clinical experiments have demonstrated that Lp(a) concentration is associated with an increased incidence of macrovascular cardiovascular disease [34]. Increased Lp(a) levels may also cause renal macrovascular disease, such as renal artery stenosis. Atherosclerotic renal artery stenosis reduces renal blood flow and is a potential cause of chronic kidney injury [35]. Furthermore, high levels of Lp(a) impair the endothelial dilatation function [36, 37], which might affect renal blood flow, thereby altering glomerular filtration pressure and leading to impaired kidney function. Finally, previous studies have confirmed that Lp(a) is rich in oxidized phospholipids, which directly cause inflammation of tissues and cells and further glomerular damage [38–41].

This study also found that individuals with higher Lp(a) levels had significantly lower TG and BMI. This may be due to the different biological properties of Lp(a) and TG. TG is substantially influenced by antihyperlipidemic drugs and lifestyle factors such as diet and physical activity. In contrast, Lp(a) is produced by liver cells and is almost completely unaffected by lipid metabolism and lifestyle factors. Therefore, we would collect more information about diet and medication history in the follow-up. BMI cannot discriminate body composition and fails to provide any indication of body fat distribution [42]. For the same reason as TG, HDL-c was higher in the high-tertile Lp(a) group. It was affected by lifestyle, such as diet, regular exercise, smoking cessation, and using some drugs [43]. Our data were consistent with the results of another study on the same ethnic group [19]. This study included more female participants than males, despite the fact that males surpass females in China, probably because the population we studied was middle-aged and older, and women live longer lives than males [44]. However, It is well established that sex hormones play an important role in kidney disease and in lipid metabolism [45, 46]. In the present study, we found no association between Lp(a) and mildly reduced eGFR in women. Considering that our population was middle-aged and older, levels of sex steroids decline significantly after menopause in women, whereas this is more gradual in men with age [47].

Several limitations of the present study should be mentioned. First, this was a cross-sectional study, so we could not draw conclusions about any causal relationship between the Lp(a) levels and mildly reduced eGFR. A longitudinal study is warranted in the future. Second, Lp(a) has multiple subtypes, and the relationship between each subtype and mildly reduced eGFR is unclear. Third, we did not have a direct measure of true kidney function and used eGFR based on the CKD-EPI formula. However, the accuracy of this formula has been verified and confirmed [25]. Fourth, our study was conducted with middle-aged and elderly Chinese participants, and further studies are needed before extrapolating our findings to other races and age groups. Lastly, whereas albuminuria was found to be strongly correlated with renal function, it was not included in our present study; hence, additional researches are required to fully remove the implications of albuminuria.

## Conclusions

Higher levels of serum Lp(a) were associated with an increased risk of mildly reduced eGFR in middle-aged and elderly Chinese people. This highlights the critical importance of evaluating and managing Lp(a) levels in the early detection of renal dysfunction.

### Supplementary Information

**Additional file 1: Supplemental Table 1.** The association between the lipoprotein(a) and risk of mildly reduced eGFR.

## Data Availability

The data utilized in this study, which was derived from a dataset produced by the Department of Endocrine and Metabolic Diseases at the First People’s Hospital of Changzhou, Third Affiliated Hospital of Soochow University, is not available to the general public due to security concerns. However, upon fair request, the corresponding author can make them available.

## Abbreviations

Lp(a): Lipoprotein(a)
CVD: Cardiovascular disease
eGFR: Estimated glomerular filtration rate
CI: Confidence interval
OR: Odds ratio
CKD: Chronic kidney disease
MI: Myocardial infarction
LDL: Low-density lipoprotein
Apo B-100: Apolipoprotein B-100
Apo(a): Apolipoprotein(a)
PDHS: Penn Diabetes Heart Study
NHANES III: Third National Health and Nutrition Examination Survey
IPAQ: International Physical Activity Questionnaire
BMI: Body mass index
SBP: Systolic blood pressure
DBP: Diastolic blood pressure
TC: Total cholesterol
TG: Triglyceride
HDL-c: High-density lipoprotein cholesterol
LDL-c: Low-density lipoprotein cholesterol
AST: Aspartate aminotransferase
ALT: Alanine aminotransferase (ALT)
Scys: Serum cystatin C
Scr: Serum creatinine
UA: Uric acid
FPG: Fasting plasma glucose
SD: Standard deviation
IQR: Interquartile range
