# Supplementary material for: Association between serum lipoprotein(a) and mildly reduced eGFR: a cross-sectional study
Source: BMC Nephrol. 2023 Dec 8;24:364. doi: 10.1186/s12882-023-03417-6 (PMC10709843; doi:10.1186/s12882-023-03417-6)
Supplement: Supplementary file 1 — Additional file 1: Supplemental Table 1. The association between the lipoprotein(a) and risk of mildly reduced eGFR. [file 12882_2023_3417_MOESM1_ESM.docx]

**Supplemental Table 1** The association between the lipoprotein(a) and risk of mildly reduced eGFR

| lipoprotein(a) levels | Case/Number(%) | OR (95%CI) | | | |
| --- | --- | --- | --- | --- | --- |
|  |  | Model 1 | Model 2 | Model 3 | Model 4 |
| Non-elevated Lp(a) | 311/639(48.7) | 1.00(Ref.) | 1.00(Ref.) | 1.00(Ref.) | 1.00(Ref.) |
| Elevated Lp(a) | 247/425(58.1) | 1.47(1.11-1.94) | 1.49(1.12-1.97) | 1.63(1.22-2.18) | 1.45(1.07-1.97) |

Model1: adjusted for age, sex, BMI;

Model2: further adjusted for current smoker (yes/no), current drinker (yes/no) and physical activity based on Model1;

Model3: further adjusted for SBP, TG, TC, LDL-c, ALT, AST ,FPG based on Model2.

Model4: further adjusted for UA based on Model3.

Abbreviations: Lp(a), lipoprotein(a); BMI, body mass index; SBP, systolic blood pressure; ALT, alanine aminotransferase; AST, aspartate aminotransferase; TC, total cholesterol; TG, triglycerides; LDL-c, low-density lipoprotein cholesterol; UA, uric acid; FPG, fasting plasma glucose
